# Hypertensive Emergency with Thrombotic Microangiopathy or TTP? A Case Series and Literature Review

**DOI:** 10.3390/jcm13071880

**Published:** 2024-03-25

**Authors:** Yang Song, Shi Yan Lee, Yen-Lin Chee, Wei-Ying Jen

**Affiliations:** 1 Department of Haematology-Oncology, National University Cancer Institute, Singapore 119074, Singapore; yen_lin_chee@nuhs.edu.sg (Y.-L.C.); jenweiying@nus.edu.sg (W.-Y.J.); 2 Division of Gastroenterology & Hepatology, Department of Medicine, National University Hospital, Singapore 119074, Singapore; shiyan.lee@mohh.com.sg

**Keywords:** TTP, MAHA, plasma exchange, thrombotic microangiopathy, hypertensive emergency, thrombotic thrombocytopenia purpura

## Abstract

Thrombotic microangiopathy (TMA) is associated with both hypertensive emergency and primary thrombocytopenia purpura (TTP). However, their clinical management is vastly different, with the latter necessitating urgent plasma exchange (PEX). We report two cases of hypertension-associated TMA (HTN-TMA) and a literature review of the clinical management of malignant hypertension. We suggest that in patients presenting with hypertensive emergency associated with TMA, a clinical diagnosis of HTN-TMA should be made, with emergent treatment to lower blood pressure started immediately. Although TTP is a differential diagnosis for TMA, PEX should not be started concurrently in the absence of other supporting evidence for TTP.

## 1. Introduction

**Figure 1:**
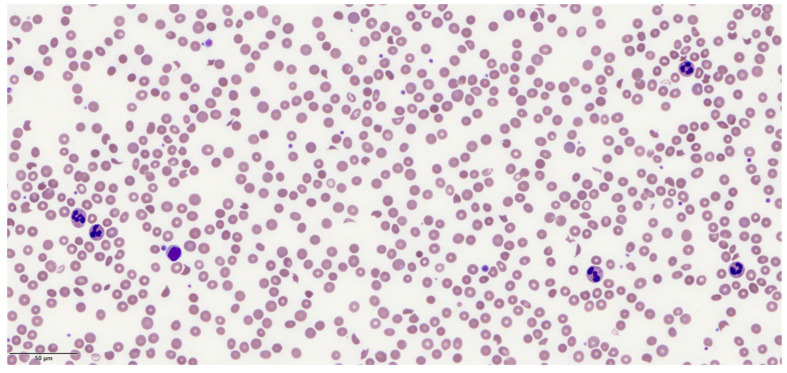
Peripheral blood film from patient 1 showing schistocytes, occasional spherocytes, and thrombocytopenia.

Hypertensive emergency, formerly known as malignant hypertension, is defined by severe hypertension associated with acute end-organ damage involving the brain, kidneys, heart, and microvasculature [1]. A proportion of patients present with thrombotic microangiopathy (TMA), characterized by a Coombs negative microangiopathic hemolytic anemia (MAHA) and thrombocytopenia (Figure 1). The pathogenesis of TMA in hypertensive emergency is thought to be due to autoregulation failure, causing endothelial injury and resulting in plasma components leaking into the vascular wall. Fibrinoid necrosis ensues, precipitating platelet and fibrin deposition. The consequent vascular lumen obliteration causes erythrocyte fragmentation, hemolytic anemia and a consumptive thrombocytopenia [2]. 

However, TMA is also associated with a diverse group of diseases, including primary thrombotic thrombocytopenia purpura (TTP), hemolytic uremic syndrome (HUS), atypical HUS, drug-induced TMA, pregnancy, and disseminated intravascular coagulation (DIC). Both hypertensive emergency with TMA (HTN-TMA) and primary TTP are considered clinical emergencies, requiring urgent treatment. Their management is vastly different, with TTP requiring urgent plasma exchange (PEX) while HTN-TMA requires immediate but careful blood pressure reduction.

TTP is a TMA that is caused by severely reduced activity of a disintegrin and metalloproteinase with a thrombospondin type 1 motif, member 13 (ADAMTS13). The diagnosis of primary TTP is supported by a severe deficiency of plasma ADAMTS13 (activity level of <10%). However, the assay is labor-intensive, and results are rarely available expediently, limiting its ability to guide immediate management. Given that the minimum criteria to diagnose HTN-TMA and primary TTP comprises only MAHA and thrombocytopenia, the differentiation between the two conditions may be challenging at initial presentation. Here, we report two cases of HTN-TMA and a literature review on the clinical management of malignant hypertension. We suggest that the diagnosis of HTN-TMA has distinct differences from primary TTP. The main focus in managing these patients should be blood pressure control, not PEX. 

## 2. Case Presentation: Patient 1 

A woman in her 60s presented to the Emergency Department with one week of dyspnoea, nausea, anorexia, and generalized abdominal discomfort. There were no other associated symptoms. She was passing urine normally and systems enquiry was unremarkable. She had a past medical history of hypertension, but had been lost to follow-up and was not taking any medication. On examination, her Glasgow Coma Scale (GCS) was 15, and she had conjunctival pallor. Fundoscopy showed normal vessels, but the fundus could not be visualized. Her temperature was 37.2 °C, heart rate 124/min, respiratory rate 24/min, and blood pressure 250/147 mmHg. Chest auscultation was normal. There was no focal neurological deficit. Laboratory tests showed hemoglobin 9.2 g/dL (11.4–14.7), MCV 90.7 fL (83.0–95.5), WBC 9.7 × 10^9^/L (3.84–10.01), platelets 116 × 10^9^/L (164–387), urea 25 mmol/L (2.0–7.5), creatinine 357 µmol/L (50–90), total bilirubin 29 µmol/L (5–30), LDH 3153 U/L (250–580), haptoglobin <30 mg/dL (30–200), PT 11.7 s (9.7–11.8), APTT 25.1 s (23.9–32.2), fibrinogen 2.54 g/L (1.79–3.98), and reticulocytes 159 × 10^9^/L (38.3–110.50). Peripheral blood film showed numerous red cell spherocytes and schistocytes (Figure 1). Electrocardiogram (ECG) showed sinus rhythm with left ventricular hypertrophy (LVH). Chest radiograph was normal. 

**Figure 2:**
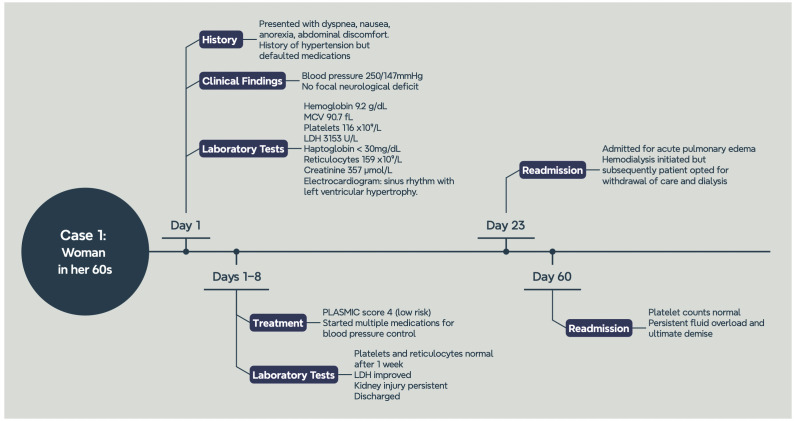
Graphical timeline of Case 1.

A diagnosis of hypertensive emergency with acute kidney injury and TMA was made. ADAMTS13 levels were not sent given the low index of suspicion for an alternative pathology. Her PLASMIC score was 4 points (low risk). Intravenous glyceryl trinitrate (GTN) was started and the patient was transferred to the general ward for further management. Multiple anti-hypertensive agents were started over the next seven days in an attempt to control her blood pressure. Her platelet counts and reticulocyte count normalized, and LDH improved after adequate blood pressure control. During her hospitalization, she was seen by the Nephrology team for raised creatinine. Ultrasound of the kidneys showed significant scarring as a sign of chronicity, and the etiology of her raised creatinine was attributed to hypertensive nephrosclerosis. Unfortunately, her renal injury was irreversible. She was subsequently discharged and her platelet count was 265 × 10^9^/L (164–387). The patient was re-admitted a few days later with acute pulmonary oedema requiring initiation of hemodialysis. During this admission, she again presented with hypertensive urgency with a blood pressure of 194/136, hemoglobin 9.5 g/dL (11.4–14.7), platelets of 81 × 10^9^/L (164–387), and reticulocyte count of 118 (38.3–110.50). Her blood pressure was controlled with medications, and her platelet count improved to 145 × 10^9^/L (164–387) a day after. Shortly after, she expressed a wish for withdrawal of care and eventually passed away during another hospital admission. Her platelet counts were normal at the time of demise (Figure 2).

## 3. Case Presentation: Patient 2

A 54-year-old man presented to Emergency Department with a one-day history of worsening headache, nausea, and epigastric pain. There was no associated fever, neck stiffness, visual disturbance, diarrhea, chest pain, or dyspnea. Systems enquiry was otherwise unremarkable. There was no significant past medical history, and he was not on any medication. On examination, his GCS was 15. His temperature was 36 °C, pulse rate 72 bpm, blood pressure 125/89 mmHg, and oxygen saturation was 99% on room air. The rest of the initial examination, including neurological system, was unremarkable. Laboratory tests showed hemoglobin 17.0 g/dL (11.4–14.7), MCV 79.8 fL (83.0–95.5), WBC 12.1 × 10^9^/L (3.84–10.01), platelets 348 × 10^9^/L (164–387), urea 4.7 mmol/L (2.0–7.5), creatinine 96 µmol/L (50–90), total bilirubin 16 µmol/L (5–30), and LDH 701 U/L (250–580). ECG was normal with sinus rhythm. 

**Figure 3:**
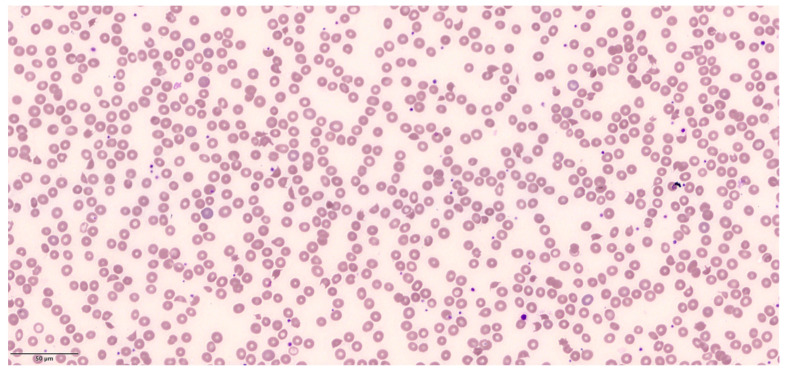
Peripheral blood film from patient 2 showing schistocytes, polychromasia, and thrombocytopenia.

**Figure 4:**
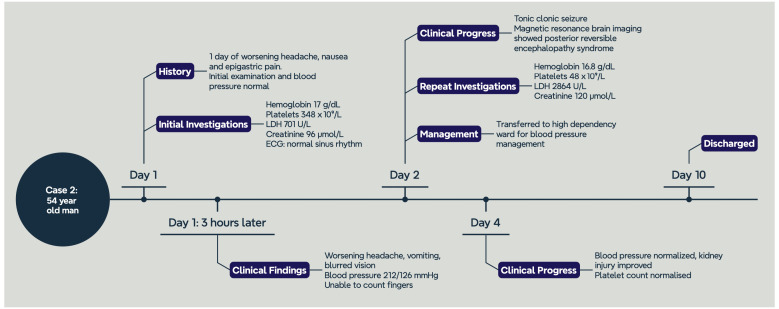
Graphical timeline of Case 2.

Three hours after admission, he complained of worsening severe headache with vomiting and blurred vision. He was unable to count fingers on examination of visual acuity. Repeat vital signs showed pulse rate 59 bpm, blood pressure 212/126 mmHg. He was unable to cooperate for fundoscopy. Amlodipine was started for his hypertension. A computed tomography (CT) head scan reported bilateral occipital hypodensities with no acute infarct. The following day, he developed a generalized tonic-clonic seizure which spontaneously aborted after a few minutes. A magnetic resonance imaging (MRI) scan of the brain showed features suggestive of posterior reversible encephalopathy syndrome (PRES). Repeat blood tests two days after admission showed hemoglobin 16.8 g/dL (11.4–14.7), WBC 14 × 10^9^/L (3.84–10.01), platelets 48 × 10^9^/L (164–387), urea 12 mmol/L (2.0–7.5), creatinine 120 µmol/L (50–90), total bilirubin 45 µmol/L (5–30), and LDH 2864 U/L (250–580). Peripheral blood film showed numerous red cell schistocytes, polychromasia, and thrombocytopenia (Figure 3). A diagnosis of hypertensive emergency with acute kidney injury, encephalopathy, and thrombotic microangiopathy was made. The blood film showed numerous red blood cell fragments with target cells and occasional microspherocytes. By this time, he was transferred to the high-dependency unit and started on intravenous GTN and labetalol. ADAMTS13 levels were not sent given the clear diagnosis of hypertensive emergency. His PLASMIC score was 5 (intermediate risk). Over the next four days, his blood pressure improved with medication titration, and his kidney injury gradually resolved. His platelet count also normalized, and he was discharged with no residual neurological deficit (Figure 4). Subsequent work-up for secondary causes of hypertension revealed a right renal artery stenosis. 

## 4. Discussion

TMA is the clinicopathological consequence of a heterogenous group of disorders encompassing autoimmune disorders, hypertension, disseminated intravascular coagulation (DIC), malignancies, and adverse drug reactions. Of these, TTP is a TMA that is caused by severely reduced activity of ADAMTS13. A lack of ADAMTS13 enzyme causes large multimers of Von Willebrand Factor (vWF) to form, causing platelet adhesion and aggregation, microthrombi formation, and end organ damage. [3] TTP can be immune mediated secondary to autoantibodies against ADAMTS13, or hereditary from mutations in the ADAMTS13 gene; the former is more common. Based on data from the Oklahoma TTP-HUS Registry, the incidence of immune TTP is approximately three per one million adults per year [4]. TTP is a medical emergency that is associated with a mortality of up to 80% if PEX is not initiated emergently. Patients with TTP usually have ADAMTS13 levels below 10%, resulting in excessive ultra-high molecular weight von Willebrand factor and microvascular thrombosis [5]. It also causes end organ damage and, although rare, a complete “pentad” of fever, thrombocytopenia, hemolytic anemia, renal dysfunction, and neurological dysfunction have been described. In contrast, patients with HTN-TMA normally have only mild deficiency of >50% ADAMTS-13 activity (van den Born 2008, Hypertension) and in the Oklahoma TTP Registry, none of the patients with malignant HTN-TMA had AMADSTS13 activity of <10% (George Blood 2010). However, in clinical practice, ADAMSTS13 has limited front-line diagnostic utility as it is a specialized laboratory test which is not routinely or immediately available in many institutions. Results of the assay also require time, and it is not routine to wait for ADAMSTS13 results before initiating PEX if the suspicion of TTP is high. Further complicating matters, PEX is not an effective treatment for secondary TMAs, and is itself associated with complications such as risks of transfusion of blood products, symptomatic electrolyte disturbance, hypovolemia, sepsis from catheter-related bloodstream infections, and complications of catheter insertion, such as hemorrhage, pneumothorax, and catheter-related thrombosis [6]. 

A reasonable threshold of diagnostic suspicion for TTP is hence required before initiating the procedure in order to avoid exposing the patient to unnecessary morbidity and mortality. This may be challenging, given that both TTP and HTN-TMA both present with fragmentation, hemolytic anemia, and thrombocytopenia. However, the first principle of making a diagnosis of TTP is to exclude secondary causes of TMA, including hypertensive emergency. Patients with TTP often do not have hypertension at presentation. A retrospective study done in France on 564 patients with TMA showed that normal BP or grade 1 hypertension (140–159/90–99 mmHg) was found in most patients with TTP [7]. Our first patient had a typical HTN-TMA presentation with history of hypertension and ECG changes suggestive of left ventricular hypertrophy. However, the second patient had neither past history of hypertension nor ECG changes. Regardless, both patients clearly had markedly elevated blood pressures with end-organ damage at presentation, making TTP unlikely.

The PLASMIC score was derived by Bendapudi and colleagues, and uses a combination of clinical history and first-line investigations to reliably predict the likelihood of severe ADAMTS13 deficiency in patients with features of TMA. The study population included all participants of a TMA registry in Boston, United States of America. All patients had thrombotic microangiopathy, thrombocytopenia, microangiopathic hemolytic anemia and an ADAMTS13 level sent. Clinical and laboratory variables were evaluated to identify those associated with severe ADAMTS13 deficiency (*p* value 0.15 or lower). These were then included in a multivariable regression model. The PLASMIC score stratified patients into low (0–4), intermediate (5) and high (6–7) risk groups. In total, >80% of patients in the high-risk group had ADAMTS13 levels <10%. This was also seen in an independent external validation cohort. In the low-risk group, 0% of patients (4% in external validation cohort) had levels <10%. For the intermediate group, performance was more variable, with 5% of patients having levels <5% (this was 24% in the external validation cohort) [8]. In our patients, the PLASMIC scores were 4 (low risk) and 5 (intermediate risk), respectively. Given the high negative predictive value of low-risk PLASMIC scores, it is reasonable to exclude TTP. Similarly, for patients at high risk of TTP, ADAMTS13 levels should be sent and PEX instituted immediately without delay, even before ADAMTS13 levels are resulted. The challenge is for patients with an intermediate risk score—the original study had a number of patients diagnosed with DIC, drug-associated TMA and atypical HUS who fell into this category. Such patients should be assessed clinically and given consideration as to how an ADAMTS13 level would change management before deciding on the most appropriate management plan. Additional evaluations for alternative explanation for the patient’s presentation should also be performed, including review of the patient’s medications, other laboratory investigations and imaging, based on clinical indication. 

Other than the PLASMIC score, the degree of thrombocytopenia and renal impairment may also point to the etiology of the TMA. A cross-sectional study performed by the French TMA center of 214 patients with TMA showed that the average platelet count was significantly lower in patients with severe ADAMTS13 deficiencies (17.4 ± 14.2 × 10^9^/L), compared with other patients in the detectable group (66.6 ± 49.3 × 10^9^/L). Mean serum creatinine levels were also significantly lower in the severe deficiency group (114 ± 68.4 µmol/L vs. 454 ± 326 µmol/L) [9]. However, while the presence of higher platelet count and serum creatinine helps support the clinical diagnosis of HTN-TMA (Patient 1), the absence of these features (Patient 2) does not mean the patient has TTP. The clinical presentation of hypertensive emergency should still take precedence as the etiology of TMA over TTP.

In both our patients, ADAMTS13 levels were not sent. This was because both patients had very high blood pressures and clinical presentations which were consistent with hypertensive emergency [10]. While it could be argued that the lack of ADAMSTS13 levels makes it difficult to prove that these were not cases of TTP, the rapid clinical improvement seen in both patients without PEX or immunosuppression makes a diagnosis of TTP extremely unlikely. The focus in management of such patients should be aggressive blood pressure control, which is required to ameliorate the risk of progression of end-organ damage. The American College of Cardiology (ACC) and American Heart Association (AHA) guidelines for the management of hypertensive emergencies recommend a reduction of blood pressure by a maximum of 25% in the first hour, followed by a target of 160/100–110 mmHg over the next 2 to 6 h, and then to normal over the next 24–48 h (excluding etiologies such as aortic dissection, severe pre-eclampsia or pheochromocytoma crisis) [11]. Sudden rapid reductions in blood pressure to normal levels are not advised, as this can result in marked reduction of blood flow to vital organs that have been accustomed to the higher level of blood pressure, leading to ischemic damage. Therefore, the recommendation is for patients to be monitored in an intensive care facility for the administration of intravenous antihypertensive medications and for close monitoring of intra-arterial pressure levels [12]. HTN-TMA generally results in a more favorable non-renal prognosis than other TMAs [13]. A retrospective study of 97 patients with malignant hypertension showed that renal impairment was more severe in patients with MAHA at presentation. However, these patients were also four times more likely to see improvement in renal function than patients without MAHA [14]. Secondary causes of hypertension should also be considered and evaluated, as they are more common in patients presenting with hypertensive emergencies [15]. They include conditions such as renal artery stenosis, hyperaldosteronism, and obstructive sleep apnea. 

## 5. Conclusions

Recognition of both thrombotic microangiopathy and clinical judgement in conjunction with validated prediction tools is essential to manage patients with HTN-TMA. Indecision may harm the patient, either from delay of therapeutic plasma exchange in TTP patients, or in treating the underlying cause for patients with secondary TMAs. 

## Data Availability

Data are not publicly available to protect patient confidentiality. Queries on the cases should be directed to the corresponding author.
